# Effects of the synbiotic composed of mangiferin and *Lactobacillus reuteri* 1–12 on type 2 diabetes mellitus rats

**DOI:** 10.3389/fmicb.2023.1158652

**Published:** 2023-04-20

**Authors:** Fanying Meng, Fan Zhang, Meng Meng, Qiuding Chen, Yaqin Yang, Wenbo Wang, Haina Xie, Xue Li, Wen Gu, Jie Yu

**Affiliations:** ^1^Yunnan Key Laboratory of Southern Medicine Utilization, College of Pharmaceutical Science, Yunnan University of Chinese Medicine, Kunming, Yunnan, China; ^2^State Key Laboratory of Medicinal Chemical Biology, College of Pharmacy and Tianjin Key Laboratory of Molecular Drug Research, Nankai University, Tianjin, China

**Keywords:** synbiotic, type 2 diabetes mellitus, *Lactobacillus reuteri* 1–12, mangiferin, autoinducer-2, intestinal flora, quorum sensing

## Abstract

Many synbiotics are effective for the prevention and treatment of type 2 diabetes mellitus (T2DM). In the treatment of T2DM, synbiotics often regulate the composition of intestinal flora, which autoinducer-2 (AI-2) may play an important role. Whether the changes of intestinal flora are related to AI-2 during synbiotics treatment of T2DM is a topic worth studying. We elucidated the effects of synbiotic composed of mangiferin and *Lactobacillus reuteri* 1–12 (SML) on T2DM rats. Male Spraque-Dawley rats were injected intraperitoneally with streptozotocin (STZ) and randomly grouped. After that, biochemical parameters, intestinal flora, fecal AI-2, and intestinal colonization of *L. reuteri* were detected. The results showed that SML had a hypoglycemic effect and mitigated the organ lesions of the liver and pancreas. Also, SML regulated biochemical parameters such as short chain fatty acids (SCFAs), lipopolysaccharides (LPS), intercellular cell adhesion molecule-1 (ICAM-1), and tumor necrosis factor-α (TNF-α). On the other hand, the proportion of probiotics, such as *Lactobacillus acidophilus*, *L. reuteri*, *Bifidobacterium pseudolongum*, *Lactobacillus murinus*, and *Lactobacillus johnsonii*, were elevated by the treatment of SML. In addition, SML promoted the colonization and proliferation of *L. reuteri* in the gut. Another thing to consider was that AI-2 was positively correlated with the total number of OTUs sequences and SML boosted AI-2 in the gut. Taken together, these results supported that SML may modulate intestinal flora through AI-2 to treat T2DM. This study provided a novel alternative strategy for the treatment of T2DM in future.

## 1. Introduction

The global prevalence of diabetes mellitus (DM) was estimated 10.5% (536.6 million people) in 2021 among people aged 20–79 years, rising to 12.2% (783.2 million) by 2045 (Sun et al., 2022). Type 2 diabetes mellitus (T2DM) is a metabolic disorder characterized by hyperglycemia caused by insulin resistance and subsequent failure of pancreatic β cells (Puddu et al., 2014), which has become a serious public health problem around the world (Qin et al., 2012).

In recent years, with the deepening of T2DM research, intestinal flora has been found to be closely related to the pathogenesis of T2DM. It has been pointed out that the analysis of intestinal flora differences can be used to predict the risk of T2DM (Karlsson et al., 2013) and guide rational drug use (Gu et al., 2017). Targeted regulation of intestinal flora may be an effective strategy to ameliorate T2DM. Besides obesity, genetics, and islet dysfunction, intestinal flora disorder may also be major factors contributing to T2DM (Ma et al., 2019). There were significant differences in the composition and abundance of intestinal flora between healthy people and T2DM patients (Hendijani and Akbari, 2018). Nowadays, the application of probiotics in the treatment of T2DM has become a hot research topic. *Bifidobacterium animalis* subsp. *lactis* 420 (Amar et al., 2011), *Lactobacillus rhamnosus* CCFM0528 (Chen et al., 2014), and *Bacillus subtilis* natto DG101 (Cardinali et al., 2020) all showed hypoglycemic effects. Probiotics are expected to become a prevention strategy for T2DM by changing intestinal flora, decreasing lipopolysaccharides (LPS), improving intestinal barrier function, and inhibiting insulin resistance (Bagarolli et al., 2017; Sun et al., 2020).

Probiotic-prebiotic combinations are called synbiotics. Many synbiotics showed better regulation effects than probiotics or prebiotics alone (Pandey et al., 2015; Kosuwon et al., 2018). The synbiotic composed of *Lactobacillus acidophilus*, *Bifidobacterium bifidum,* and oligofructose could reduce blood glucose and increase high-density lipoprotein cholesterol in T2DM patients (Moroti et al., 2012). Synbiotic yogurt containing monk fruit extract could improve blood glucose regulation, reduce insulin resistance and glycosylated hemoglobin, improve short chain fatty acids (SCFAs) level and intestinal microbiota status, and ameliorate liver and kidney damage in T2DM rats (Ban et al., 2020). One clinical study showed that synbiotic (*Lactobacillus sporogenes*, inulin, and β-carotene) had favorable effects on insulin, HOMA-IR, HOMA-B, triglycerides, VLDL-cholesterol, total-/HDL-cholesterol ratio, NO, and GSH levels (Asemi et al., 2016). Many studies have proved the well therapeutic effect of synbiotics without any adverse reactions (Chiou et al., 2021; Naseri et al., 2022; Zhao et al., 2022).

Generally speaking, synbiotics enhanced therapeutic effect comes from the fact that prebiotics can promote the proliferation of probiotics, make probiotics the dominant species, and improve intestinal microecology (Nyangale et al., 2014; Kanjan and Hongpattarakere, 2017; Sarao and Arora, 2017). Quorum sensing (QS) is the communication language between microorganisms. When the bacterial density reaches a certain level, bacteria regulate their group behaviors by secreting, recognizing, and responding to automatic inducers (AIs; Waters and Bassler, 2005). Some studies have found that increased autoinducer-2 (AI-2), as a kind of AIs, could regulate the intestinal flora disorder caused by antibiotics at the phylum level (Sun et al., 2015; Thompson et al., 2015). QS may be related to the composition and metabolic changes of intestinal flora, and intestinal AI-2 is likely to be a simple, rapid, inexpensive, and non-invasive indicator to detect the diversity of intestinal flora.

We previously isolated a *Lactobacillus reuteri* from a healthy Sprague–Dawley rat and named it *L. reuteri* 1–12. Several studies have shown that *L. reuteri* has a good therapeutic effect in DM (Simon et al., 2015; Hsieh et al., 2018; Chiang et al., 2021). Moreover, we have found that mangiferin as the S-ribosylhomocysteinase (LuxS) inhibitor can significantly promote the proliferation of *L. reuteri* 1–12 (Meng et al., 2022). Meanwhile, mangiferin was notably present in mango fruits and mango by-products (Berardini et al., 2005; Dorta et al., 2014), and *L. reuteri* can be added to food (Garde et al., 2016). Based on the above situation, we attempted to study the therapeutic effect of synbiotic composed of mangiferin and *L. reuteri* 1–12 (SML) on T2DM rats, evaluate the therapeutic effect, explore the mechanism, and clarify the value of SML in regulating intestinal flora.

## 2. Materials and methods

### 2.1. Bacterial strains and reagents

*Lactobacillus reuteri* 1–12 (Meng et al., 2022) and *Lactobacillus faecalis* 2–84 (Yang et al., 2021) were isolated from the healthy rat intestine and identified via 16S rRNA sequencing. *Lactobacillus plantarum* came from Professor Yiyong Luo of Kunming University of Science and Technology. The reporter strain *Vibrio harveyi* BB170 (strain BNCC337376) was purchased from Beijing Beina Biological Co., Ltd. Mangiferin was obtained from Shanghai Yuanye Bio-Technology Co., Ltd. (purity > 95%). Streptozocin (STZ) was purchased from Beijing Solarbio Science and Technology Co., Ltd. Metformin hydrochloride tablets were obtained from Sino-American Shanghai Squibb Pharmaceuticals Ltd. Commercial enzyme-linked immunosorbent assay (ELISA) kits were used for the quantifications of fasting insulin (FINS), glucagon, SCFAs, LPS, intercellular adhesion factor 1 (ICAM-1), tumor necrosis factor α (TNF-α), interferon γ (IFN-γ), interleukin 2 (IL-2), and interleukin 6 (IL-6).

### 2.2. Animals experiments

Forty-eight healthy male Sprague–Dawley rats (180 ± 20 g) were purchased from Hunan SJA Laboratory Animal Co., Ltd. The experimental conditions and methods were reviewed and qualified by the Ethics Committee of Animal Protection and Experimental system of the Yunnan University of Chinese Medicine. Experimental animals were fed under the same conditions, eating and drinking freely. They were kept in a cage room (12 h light/dark cycle, at 23–27°C and 30–60% relative humidity). For adaptation, all rats were enrolled in a familiarization period for 1 week.

Preparation for medicines: *Lactobacillus reuteri* 1–12 was cultured in MRS medium under anaerobic, 37°C, 180 rpm conditions. Then, centrifuged at 4°C for 10 min at 5,000 rpm. After that, washed bacterial sludge twice with PBS, and added 30% sucrose solution and mixed well. Subsequently, the total living bacteria was adjusted to 5 × 10^10^ CFU/ml and stored at − 80°C. Finally, the bacterial solution was diluted 10 times before intragastric administration. Furthermore, mangiferin and metformin hydrochloride tablets were dissolved in 3% sucrose solution.

The modeling method of T2DM rats was as follows: after fasting for 16 h, rats were intraperitoneally injected with 40 mg/kg STZ (dissolved in citric acid-sodium citrate buffer solution, placed on ice and avoided light, and used immediately after preparation). One week later, blood was collected from the tail tip to measure fasting blood glucose (FBG) by blood glucose meter (Roche, China), and FBG ≥ 11.1 mmol/l was used as the T2DM standard.

Then, the T2DM rats were randomly divided into the T2DM model group (MOD), *L. reuteri* 1–12 group (Lre), low-dose mangiferin group (MGFL), high-dose mangiferin group (MGFH), low-dose mangiferin + *L. reuteri* 1–12 group (SML-L), high-dose mangiferin + *L. reuteri* 1–12 group (SML-H), metformin hydrochloride group (MET), and rats without STZ injection were set as the control group (CON; *n* = 6 rats per group). During the modeling of T2DM, CON was intraperitoneally injected with 0.1 mol/l sodium citrate buffer. Medicines were given from the successful T2DM model to the end of the experiment (which lasted 6 weeks). Treatment methods for each group are shown in Table 1. In addition, high-fat and high-sugar diet (HF-HSD) was prepared by 67% standard diet, 10% lard, 20% sucrose, 2.5% cholesterol, and 0.5% sodium cholate.

**Table 1:** Treatment methods of rats in each group.

Groups	STZ	Diet	Daily administration
CON	NO	Standard diet	2 ml 3% sucrose solution
MOD	YES	HF-HSD	2 ml 3% sucrose solution
Lre	YES	HF-HSD	1 ml *L. reuteri* 1–12 + 1 ml 3% sucrose solution
MGFL	YES	HF-HSD	1 ml 20 mg/kg mangiferin+1 ml 3% sucrose solution
MGFH	YES	HF-HSD	1 ml 60 mg/kg mangiferin+1 ml 3% sucrose solution
SML-L	YES	HF-HSD	1 ml *L. reuteri* 1–12 + 1 ml 20 mg/kg mangiferin
SML-H	YES	HF-HSD	1 ml *L. reuteri* 1–12 + 1 ml 60 mg/kg mangiferin
MET	YES	HF-HSD	1 ml 90 mg/kg metformin hydrochloride + 1 ml 3% sucrose solution

### 2.3. Detection of pharmacodynamic indexes of SML on T2DM rats

#### 2.3.1. Fasting blood glucose and oral glucose tolerance test (OGTT) measurement

FBG was measured 1 week after STZ injection and 6 weeks after drug intervention. Fasting for 12 h before measurement, tail vein blood was taken and measured.

Oral glucose tolerance test was measured two days before the rats were sacrificed. All groups were fasted for 12 h and then given intragastric administration according to Table 1. One hour later, all rats were given 2 g/kg glucose solution (500 mg/ml) intragastric. FBG was measured at 0, 30, 60, 90, and 120 min.

#### 2.3.2. Collection and determination of serum biochemical parameters

At the end of this experiment, the rats were anesthetized with pentobarbital sodium, and blood was taken from the abdominal aorta. The blood was left standing at room temperature for 30 min and centrifuged at 3,500 rpm for 15 min at 4°C, and serum was collected and stored at −80°C. FINS, glucagon, SCFAs, LPS, ICAM-1, TNF-α, IFN-γ, IL-2, and IL-6 were determined by ELISA.

#### 2.3.3. Histopathological observation

After the rats were sacrificed, the liver and pancreas tissues were immersed in 4% paraformaldehyde solution, embedded in paraffin, and stained with hematoxylin–eosin. The micrographs of the liver and pancreas were evaluated at 200 × magnification, and three animals in each group were observed under the microscope in four fields of view.

### 2.4. 16S rRNA gene sequence analysis

After the animals killed, the cecum contents of each group were taken and quickly freezed with liquid nitrogen. Three independent samples from each group were selected for determination.

Microbial community genomic DNA was extracted from cecal contents samples using the E.Z.N.A.^®^ soil DNA Kit (Omega Bio-tek, Norcross, GA, United States) according to the manufacturer’s instructions. The hypervariable region V3–V4 of the bacterial 16S rRNA gene was amplified with primer pairs 338F (5′-ACTCCTACGGGAGGCAGCAG-3′) and 806R (5′-GGACTACHVGGGTWTCTAAT-3′) by an ABI GeneAmp^®^ 9,700 PCR thermocycler (ABI, CA, United States). The amplification procedure was as follows: initial denaturation at 95°C for 3 min, followed by 27 cycles of denaturing at 95°C for 30 s, annealing at 55°C for 30 s and extension at 72°C for 45 s, and single extension at 72°C for 10 min. Purified amplicons were pooled in equimolar and paired-end sequenced on an Illumina MiSeq PE300 platform (Illumina, San Diego, United States) according to the standard protocols by Majorbio Bio-Pharm Technology Co. Ltd. (Shanghai, China). The raw reads were deposited into the NCBI Sequence Read Archive (SRA) database (Accession Number: SRP373646).

We first used the fastp (Chen et al., 2018; https://github.com/OpenGene/fastp, version 0.20.0) to quality of original sequence, then used FLASH (Magoč and Salzberg, 2011; http://www.cbcb.umd.edu/software/flash, version 1.2.7) for sequence assembly. After that, according to the similarity of 97%, UPARSE (Edgar, 2013; http://drive5.com/uparse/, version 7.1) was used for operational taxonomic units (OTU) clustering. Finally, taxonomic annotation of OTU species was performed by using RDP classifier (Wang et al., 2007; http://rdp.cme.msu.edu/, version 2.11) compared with Silva 16S rRNA gene database (v138), where the confidence threshold was 70%.

All data analyses were performed using the platform of Majorbio^1^. Mothur (Schloss et al., 2009)^2^ was used to calculate the Alpha diversity, such as Chao 1, Shannon index, and used the Wilcoxon rank-sum test for Alpha diversity analysis of the differences between groups. Principal coordinate analysis (PCoA) was used to test the similarity of microbial community structure among samples based on Bray–Curtis distance algorithm, and PERMANOVA nonparametric test was used to analyze whether the differences in microbial community structure between sample groups were significant. Linear discriminant analysis Effect Size (LEfSe; Segata et al., 2011)^3^ identified microorganisms with significant differences in level abundance from phylum to genus among different groups. The effect of biochemical parameters on intestinal flora community structure was investigated by distance-based redundancy analysis. In addition, based on Spearman, species were selected for correlation network graph analysis (Barberán et al., 2012).

### 2.5. Detection of fecal AI-2

Two days before the end of the experiment, the feces of rats (*n* = 6) in each group were taken and stored in liquid nitrogen rapid cooling and then transferred to a − 80°C refrigerator for storage.

The method of Thompson et al. (2015) was used for modification. Concisely, the feces (*n* = 48) were individually frozen with liquid nitrogen and ground up. Then, 0.2 g of feces was precisely weighed and mixed with 0.1 M MOPS solution (pH = 7) in a ratio of 1:10, and the feces were homogenized. After that, the solutions were centrifuged at 12,000 rpm at 4°C for 10 min, and the feces were freeze-dried after adding the same amount of methanol. Finally, the feces were suspended with two times sterile water and filtered by 0.22 μm microporous membrane. Sterile water was used as the negative control.

At 180 rpm and 30°C, the *V. harveyi* BB170 was cultured overnight in 2216E medium, and the OD_600_ value reached 0.8. A partial bacterial solution was taken and diluted 5,000 times with fresh 2216E medium. Filtrate of feces and sterile water were mixed with diluted *V. harveyi* BB170 in a ratio of 1:9, respectively, and incubated at 180 rpm at 30°C for 1 h. After that, bacteria solution was added to 96-well black plates (200 μl/well), and set 3 multiple wells for each sample. Varioskan flash automatic microplate reader (Thermo Scientific, United States) was used to determine the bioluminescence. Finally, the relative luminescence units (RLU) of AI-2 was calculated (RLU = sample mean luminescence/negative control mean luminescence).

### 2.6. Absolute quantification of *Lactobacillus reuteri*

#### 2.6.1. DNA extraction

*Lactobacillus reuteri* 1–12, *L. faecalis* 2–84 and *L. plantarum* were used to test the specificity of the primers. Herein, we needed to extract the DNA of these three bacteria. *L. reuteri* 1–12 and *L. faecalis* 2–84 under anaerobic conditions, *L. plantarum* was not subjected to anaerobic conditions. Then, the each bacteria was cultured overnight at 180 rpm, 37°C until the OD_600_ reached 0.8. Next, the DNA was extracted according to the instructions of the TIANamp Bacteria DNA Kit (Tiangen Biotech, China).

Fecal DNA was used for absolute quantification of *L. reuteri*. The feces (*n* = 48) were grinded with liquid nitrogen, and set three replicates per sample. Subsequently, the DNA was extracted according to the instructions of the M5 Stool Genomic Plus DNA Kit (Mei5 Biotechnology, Co., Ltd).

#### 2.6.2. Primers design

DNAMAN was used to compare the 16S rRNA sequences of *L. reuteri* and other *Lactobacillus* strains to search for conserved regions within species. Primer 5.0 was used to design the primers. Also, some primers were derived from other literatures (Song et al., 2000; Qin et al., 2020).

#### 2.6.3. Primers specificity verification

The DNA of *L. reuteri* 1–12, *L. faecalis* 2–84, and *L. plantarum* was amplified by PCR. The PCR reaction system (25 μl) contained: 2 × Taq Master Mix (12.5 μl), ddH_2_O (8.5 μl), F primer (1 μl), R primer (1 μl), and DNA (2 μl). The PCR amplification condition was initial denaturation at 95°C for 5 min, followed by 30 cycles of 95°C 30 s, 56°C 30 s, 72°C 12 s, and then a final extension at 72°C for 5 min. Then, PCR products were detected by electrophoresis in TBE gel containing 2% agarose.

#### 2.6.4. Establishment of the standard curve

According to the instructions of the DNA Gel Extraction Kit (Beyotime, China), PCR products of *L. reuteri* 1–12 target genes were purified to obtain DNA. Then, DNA concentration was measured, and a 10-fold serial dilution was performed. After that, the absolute copy number of DNA per 200 mg of feces was calculated. Finally, the diluted DNA samples were amplified by quantitative PCR (qPCR) to establish a standard curve. Specifically, the reaction system (20 μl) of qPCR contained: 2 × SYBR Green PCR Master Mix (10 μl), ddH_2_O (7.2 μl), F primer (0.4 μl), R primer (0.4 μl), and DNA (2 μl). The qPCR amplification conditions were: (i) 95°C for 5 min and (ii) 45 cycles at 95°C for 30 s, 58°C for 60 s, and 72°C for 90 s.

#### 2.6.5. The qPCR detection

Fecal DNA were detected by the above qPCR method. Then, the absolute copy number was calculated according to the standard curve.

### 2.7. Statistical analysis of data

The experimental data were expressed by mean ± standard deviation and analyzed and processed by GraphPad Prism 8 statistical software. All data were analyzed by one-way ANOVA or two-way ANOVA, and the *p < 0.05, **p < 0.01, the ***p < 0.001, ****p < 0.0001 was considered statistically significant.

## 3. Results

### 3.1. Effects of SML on blood glucose metabolism in T2DM rats

#### 3.1.1. Effects of SML on FBG in T2DM rats

**Figure 1:**
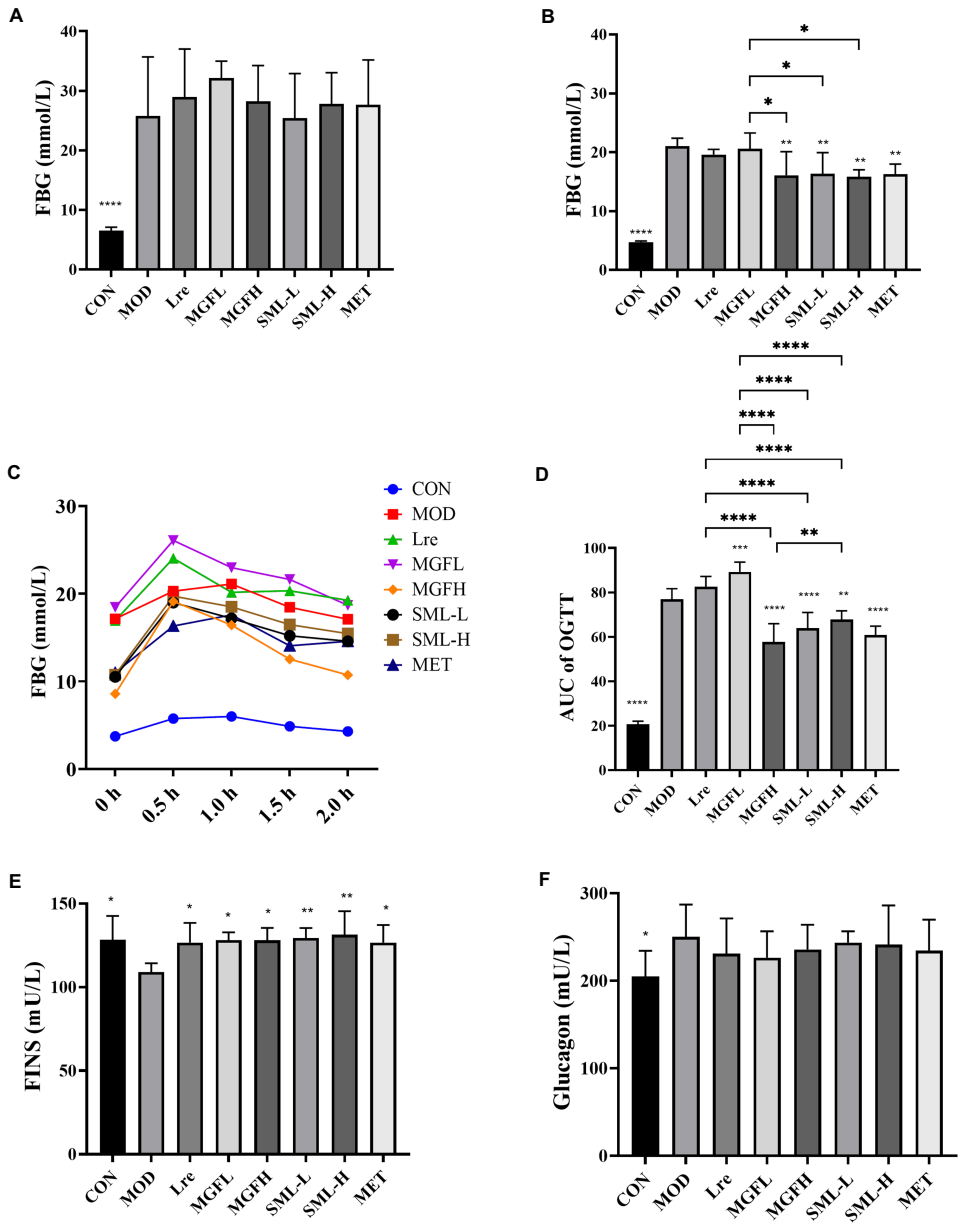
Effects of SML on glucose metabolism. **(A,B)**, respectively, represented the FBG level before and after SML intervention. **(C)** was the OGTT curve, and **(D)** was the AUC of OGTT. **(E,F)**, respectively, showed FINS and glucagon levels. ^*^p < 0.05, ^**^p < 0.01, ^***^p < 0.001, ^****^p < 0.0001 compared with the MOD, and the SML and its components were compared.

The body weight decreased and food intake increased of T2DM rats (Supplementary Figure S1). One week after STZ injection, there was no significant difference in FBG among Lre, MGFL, MGFH, SML-L, SML-H, MET, and MOD (Figure 1A). The day before the rats were killed, MGFH (^**^p < 0.01), SML-L (^**^p < 0.01), SML-H (^**^p < 0.01), and MET (^**^p < 0.01) FBG level were significantly lower than MOD (Figure 1B). Moreover, SML (including both SML-L and SML-H) had better hypoglycemic effect than MGFL (Figure 1B). Furthermore, the hypoglycemic effect of SML was similar to MET.

#### 3.1.2. Effects of SML on OGTT In T2DM rats

After giving the rats glucose solution, the FBG increased sharply and reached the peak within 0.5–1 h. And the FBG decreased nearly to the initial level with the time prolongation up to 2 h. During the whole process, the FBG of MOD rats was higher than that of CON at every time point. OGTT curves of Lre and MGFL were higher than MOD, while OGTT curves of MGFH, SML-L, SML-H and MET were lower than MOD (Figure 1C). Subsequently, the area under curve (AUC) of OGTT was calculated. AUC of OGTT had no significant difference between the Lre and the MOD (*p* > 0.05). MGFL significantly increased (*p* < 0.001), but CON (*p* < 0.0001), MGFH (*p* < 0.0001), SML-L (*p* < 0.0001), SML-H (*p* < 0.01), MET (*p* < 0.0001) significantly decreased (Figure 1D). Compared with *L. reuteri* 1–12 and mangiferin administration alone, SML could better ameliorate the abnormal glucose tolerance (Figure 1D).

#### 3.1.3. Effects of SML on insulin sensitivity in T2DM rats

Compared to the MOD, CON (*p* < 0.05), Lre (*p* < 0.05), MGFL (*p* < 0.05), MGFH (*p* < 0.05), SML-L (*p* < 0.01), SML-H (*p* < 0.01), and MET (*p* < 0.05) significantly increased the FINS (Figure 1E). Nevertheless, there was no significant difference in glucagon content among T2DM rats (Figure 1F). However, SML did not show an advantage.

### 3.2. Effects of SML on serum biochemical parameters of T2DM rats

**Figure 2:**
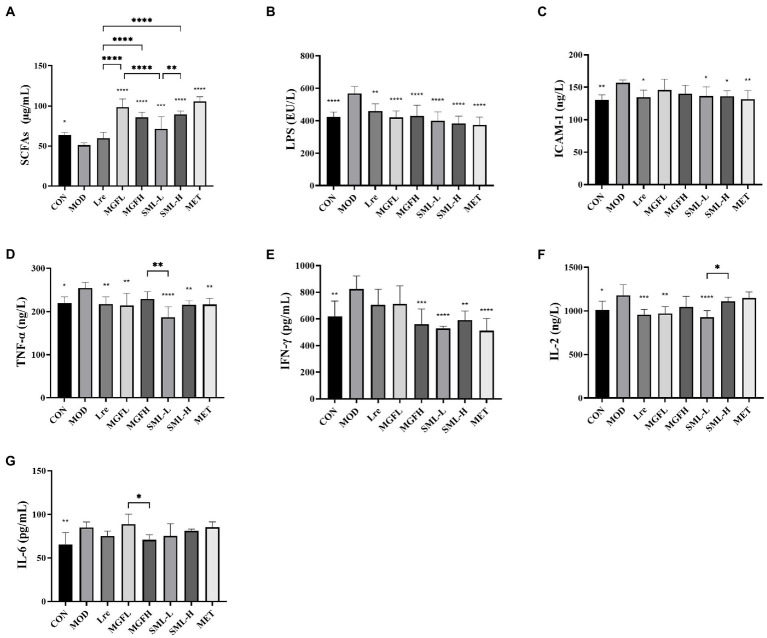
Serum biochemical parameters in T2DM rats. **(A–G)** represented the contents of SCFAs, LPS, ICAM-1, TNF-α, IFN-γ, IL-2, and IL-6. ^*^p < 0.05, ^**^p < 0.01, ^***^p < 0.001, ^****^p < 0.0001 compared with the MOD, and the SML and its components were compared.

Serum biochemical parameters of rats in each group were detected. SML increased serum SCFAs and decreased LPS, ICAM-1, TNF-α, IFN-γ, and IL-2 (Figures 2A–F). However, there was no significant difference in IL-6 between groups (Figure 2G). Interestingly, the differences between SML and its components in regulating biochemical parameters were not particularly significant.

### 3.3. Effects of SML on histopathology of liver and pancreas in T2DM rats

**Figure 3:**
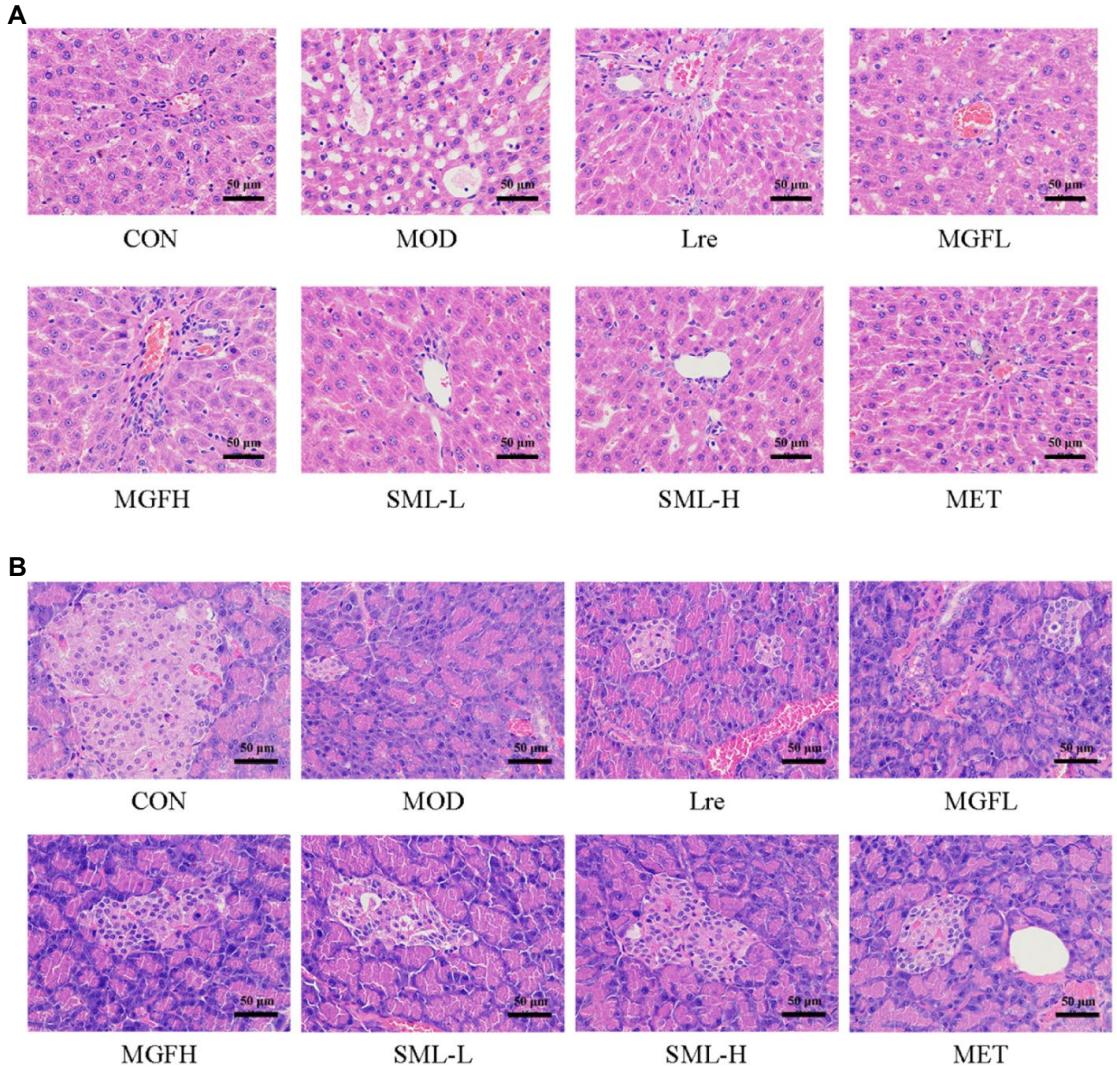
Photomicrographs. **(A,B)** were representative photomicrographs of the liver and pancreas, respectively (original magnification 200 ×).

As can be seen from Figure 3A, the liver tissue structure of rats in CON was complete, the liver cords were clear, and there was no steatosis and vacuolar degeneration. The cells in the MOD were disordered, presenting diffuse steatosis, vacuoles of different sizes, and liver cords were disordered. Hepatic steatosis and hepatic cord disorder were ameliorated in the administration group. Each administration group showed some amelioration upon MOD. Among them, the SML had less vacuolar degeneration and more regular hepatic cord arrangement, and the effect was more potent than that of utilization alone.

Hematoxylin–eosin staining of the pancreas is shown in Figure 3B. The number and volume of islets in the CON were normal, the islet masses were round or oval with clear boundaries, and the cells in the islets were regular and abundant cytoplasm. In MOD, the number of cells in the islet was significantly reduced, with irregular shape and unclear capillary structure. Obviously, there were more islet cells, less necrosis of islet cells, mild atrophy of islet, more regular shape, clearer boundary, and richer cytoplasm in SML. These results suggested that SML can mitigate the organ lesions of the liver and pancreas in T2DM rats to a certain extent.

### 3.4. Analysis of intestinal flora in T2DM rats

#### 3.4.1. Effects of SML on intestinal flora abundance and diversity in T2DM rats

OTU refer to unified markers artificially set for a taxon to facilitate analysis in phylogeny or population genetics studies. As can be seen from Table 2, the abundance and diversity of SML-L and MOD were highest, while MET was lowest. After comprehensive analysis, the abundance and diversity of each group were ranked as follows: SML-L / MOD > MGFH > SML-H / CON > MGFL / Lre > MET.

**Table 2:** Abundance and diversity indices of microbial communities.

Groups	Sobs	Shannon	Simpson	Ace	Chao
CON	228.67	3.50	0.06	255.27	255.07
MOD	249.67	3.69	0.05	274.89	280.49
Lre	216.33	3.16	0.10	250.05	254.85
MGFL	214.67	3.40	0.06	235.40	236.19
MGFH	240.33	3.37	0.08	271.29	277.98
SML-L	257.67	3.63	0.06	283.37	290.40
SML-H	229.33	3.42	0.07	255.89	257.30
MET	171.67	2.99	0.10	201.22	195.27

#### 3.4.2. Effects of SML on the composition of intestinal flora in T2DM rats

**Figure 4:**
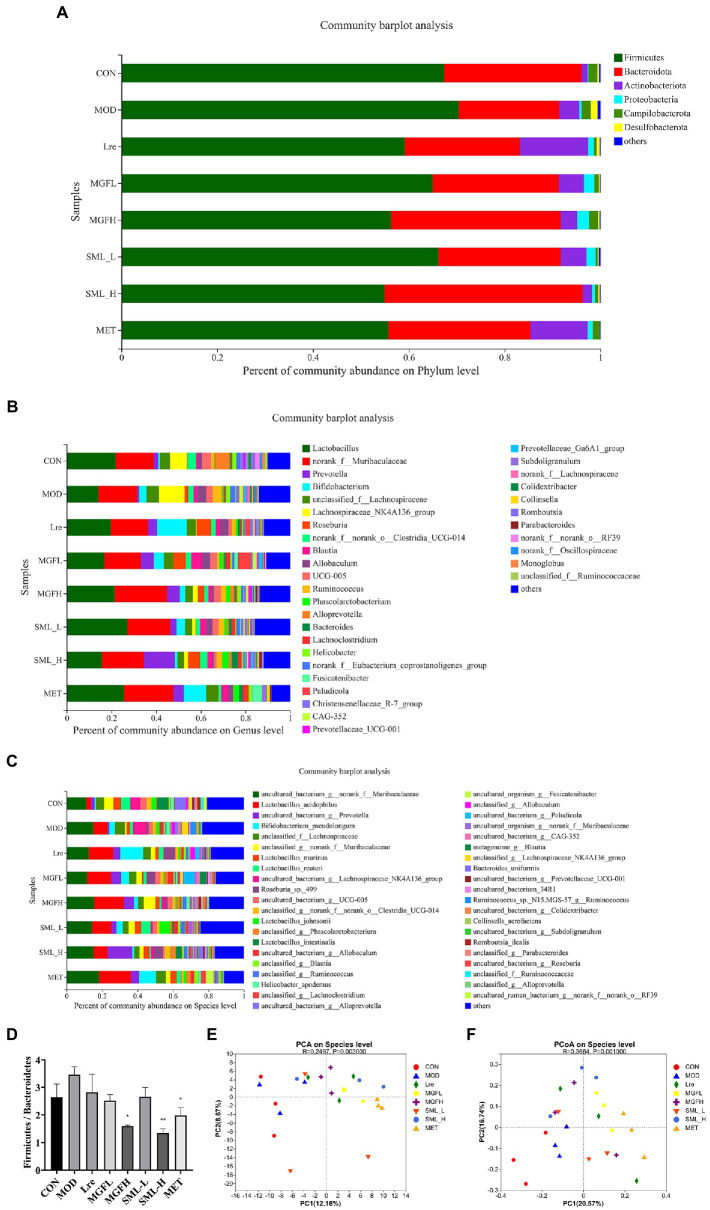
Composition of intestinal flora. **(A–C)**, respectively, showed intestinal flora relative proportion at phylum, genus, and species levels. **(D)** was the proportion of Firmicutes/Bacteroidetes. **(E,F)** were PCA and PCoA, respectively. The distance between points indicated the correlation between samples. ^*^p < 0.05, ^**^p < 0.01 compared with the MOD.

Sequencing results showed that microbes were mainly classified into Bacteroidetes, Firmicutes, Actinobacteriota, and Proteobacteria at the phylum level. Compared with MOD, CON, Lre, MGFL, MGFH, SML-L, SML-H, and MET had a lower proportion of Firmicutes and a higher proportion of Bacteroidetes (Figure 4A). Moreover, the ratio of Firmicutes/Bacteroidetes in MGFH (*p* < 0.05), SML-H (*p* < 0.01), and MET (*p* < 0.05) was significantly lower than that in MOD (Figure 4D). Each group had significant differences in intestinal flora at genus and species levels (Figures 4B,C). Importantly, the proportion of *L. reuteri* was significantly reduced in the MOD, while SML-L significantly increased.

Principal component analysis (PCA) and PCoA were performed. The results showed that CON and MOD were not well distinguished, and the intestinal flora was more different after drug administration (Figures 4E,F).

#### 3.4.3. Analysis of intestinal flora difference in T2DM rats

**Figure 5:**
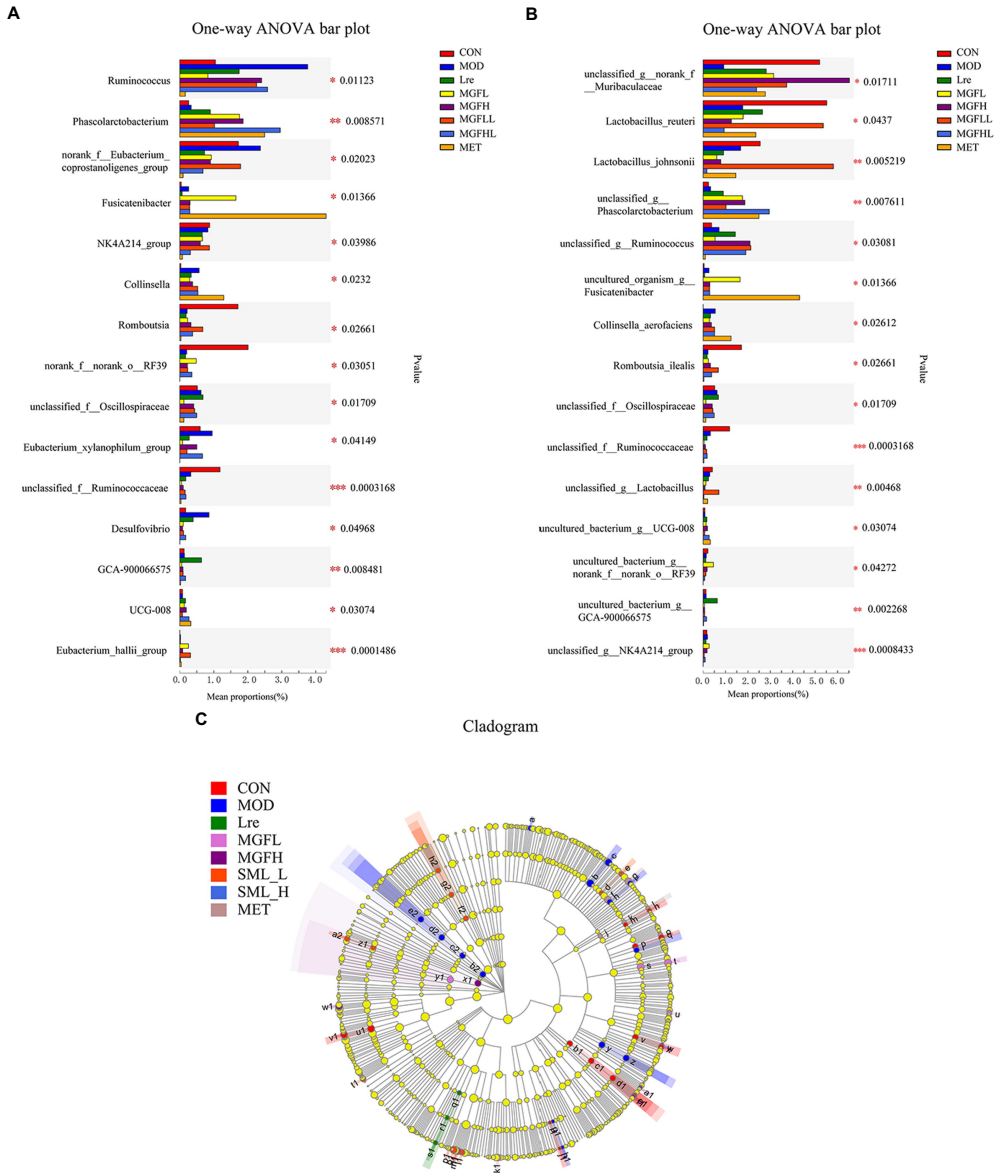
Analysis of intestinal microbiome differences. **(A,B)** were the analysis results of the differences between groups at the genus and species levels. **(C)** represented the LEfSe analysis (the circle of evolutionary branching from internal to external radiation represented the classification level from phylum to species), and different color nodes represented microbial groups that were significantly enriched in corresponding groups and had a significant influence on the differences between groups. The yellowy nodes indicated the microbial groups that had no significant difference among different groups.

Compared with MOD, other groups can reduce the proportion of *Ruminococcus* and *Desulfovibrio*, but increase the proportion of *Phascolarctobacterium* (Figure 5A). Another result showed that the proportion of *Collinsella* and *Fusicatenibacter* increased in the MET, while *Romboutsia* decreased (Figure 5A). There were also significant differences at the species level, and it was statistically significant (Figure 5B). *Lactobacillus reuteri*, *Lactobacillus johnsonii*, and *Romboutsia ilealis* accounted for a low proportion in MOD, while the proportion of *Lactobacillus reuteri* and *Lactobacillus johnsonii* increased effectively in SML-L.

The LEfSe results showed that there were significant differences in intestinal flora at different classification levels (LDA > 3; Figure 5C). There were 15, 15, 3, 5, 4, 10, and 4 microbial groups that played a potential important role in CON, MOD, Lre, MGFL, MGFH, SML-L, and MET, respectively. Even more to the point, LEfSe analysis results showed that *Lactobacillus reuteri* played a key role in maintaining healthy intestinal microecology in CON and was one of the biomarkers.

#### 3.4.4. Association analysis of SML on intestinal bacteria and biochemical parameters in T2DM rats

**Figure 6:**
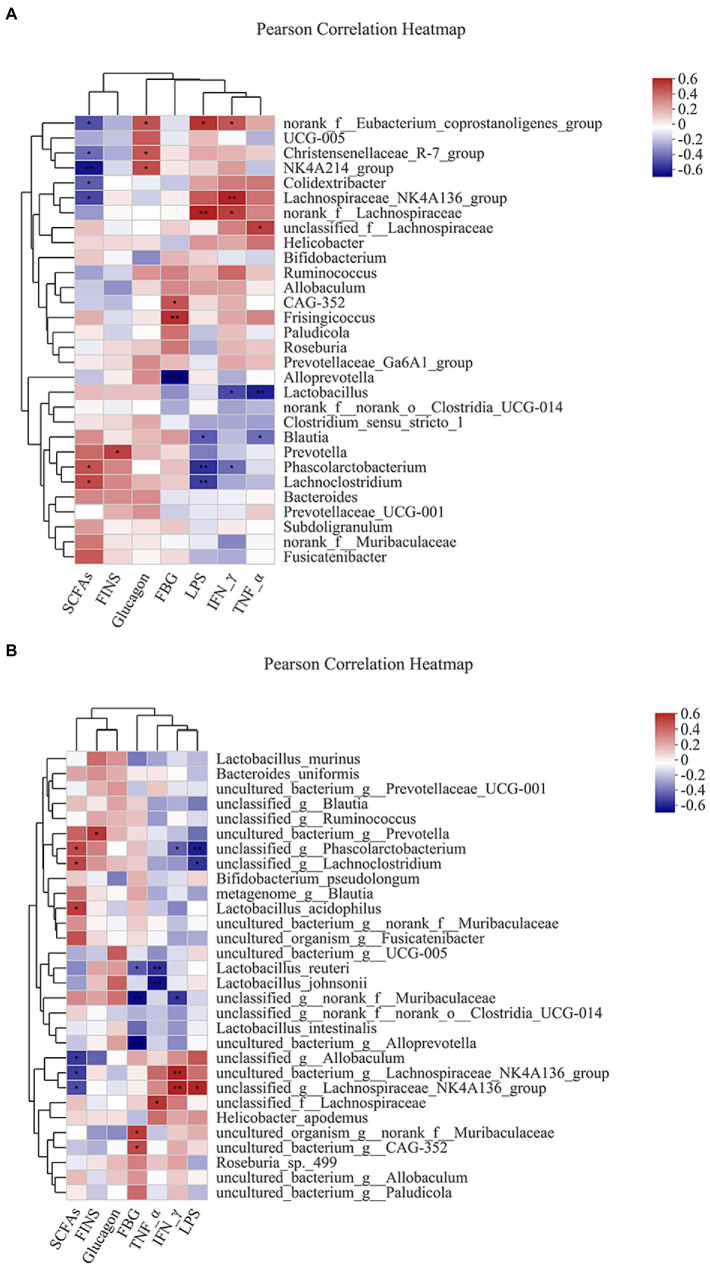
Heatmap of correlation analysis between intestinal bacteria and biochemical parameters. **(A,B)** represented the correlation analysis between intestinal bacteria and biochemical parameters at the genus and species levels. ^*^p ≤ 0.05, ^**^p ≤ 0.01, ^***^p ≤ 0.001.

Pearson analysis was used to visually demonstrate the correlation between intestinal bacteria and biochemical parameters. The results showed that the genus level of intestinal bacteria was closely related to biochemical parameters (Figure 6A). *Phascolarctobacterium* and *Lachnoclostridium* were positively correlated with SCFAs. *Prevotella* was positively correlated with FINS. *Dubosiella* was positively correlated with glucagon. *Frisingicoccus* was positively correlated with FBG, while *Alloprevotella* was negatively correlated with FBG. *Blautia* and *Phascolarctobacterium* were negatively correlated with LPS. *Lactobacillus* and *Phascolarctobacterium* were negatively correlated with IFN-γ. The *Lactobacillus* and *Blautia* were negatively correlated with TNF-α.

Similarly, species levels of intestinal bacteria were associated with biochemical parameters (Figure 6B). The *Lactobacillus acidophilus* was positively correlated with SCFAs. *Lactobacillus reuteri* was negatively correlated with FBG. *Lactobacillus reuteri* and *Lactobacillus johnsonii* were negatively correlated with TNF-α.

#### 3.4.5. Effects of SML on the proportion of SCFA-producing bacteria in T2DM rats

**Figure 7:**
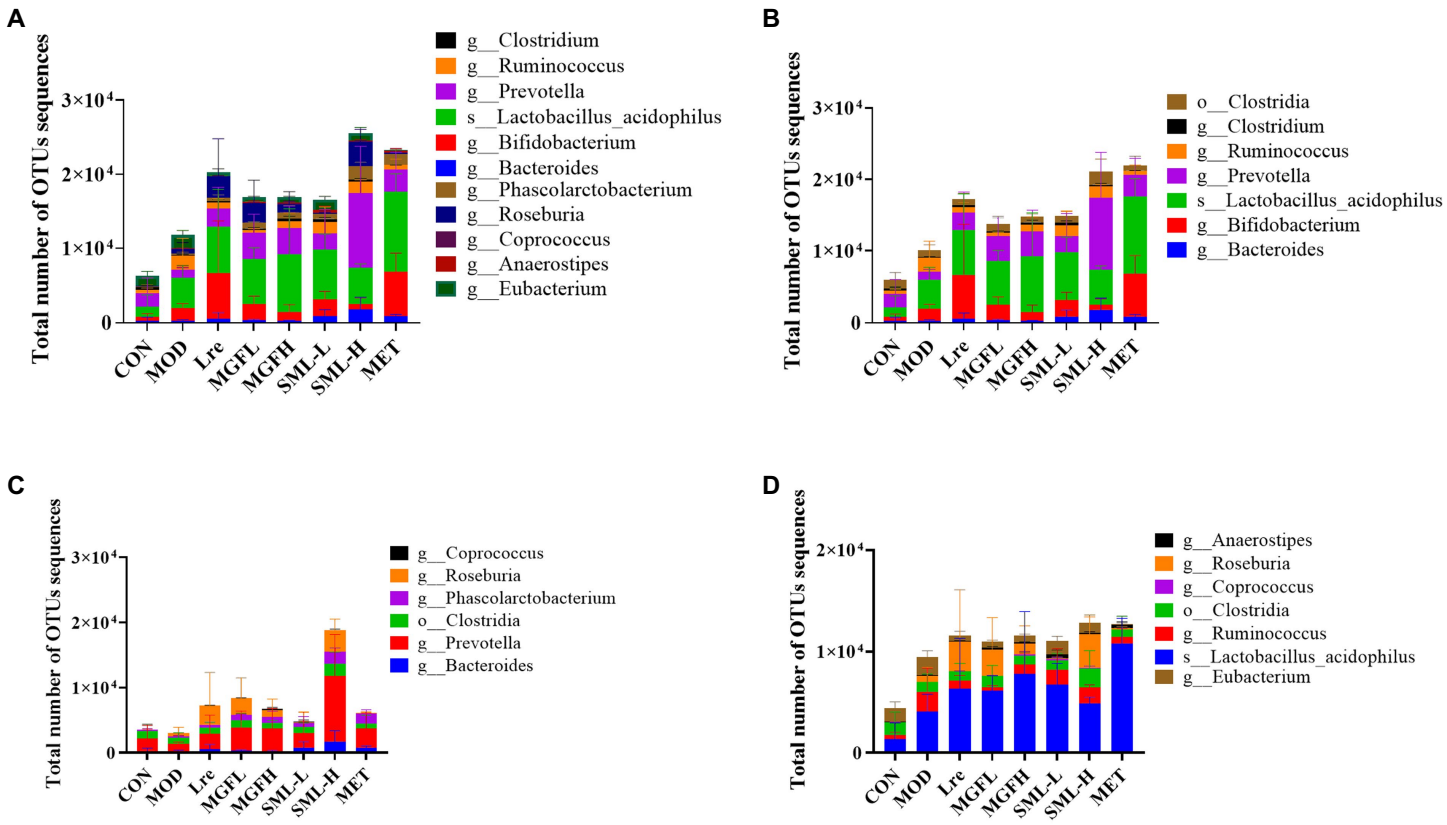
Total number of OTUs sequences statistics of SCFA-producing bacteria **(A–D)** were the results of SCFA-producing bacteria, acetic acid-producing bacteria, propionic acid-producing bacteria, and butyric acid-producing bacteria in the intestines, respectively.

The proportion of SCFA-producing bacteria was analyzed by referring to the literatures (Macfarlane and Macfarlane, 2012; Louis et al., 2014; Koh et al., 2016; LeBlanc et al., 2017), and the specific results in this study were presented (Figure 7). Briefly, acetogenic bacteria mainly include *Bacteroides*, *Bifidobacterium*, *Lactobacillus acidophilus*, etc., while propionic acid-producing bacteria mainly include *Prevotella*, *Phascolarctobacterium*, *Clostridia*, etc. Butyric-producing bacteria mainly include *Lactobacillus acidophilus*, *Eubacterium*, *Ruminococcus,* and so on. Compared with CON, the proportion of acetic acid-producing bacteria and butyric-producing bacteria increased in MOD, especially *Bifidobacterium*, *Lactobacillus acidophilus*, *Ruminococcus*, etc. On the other hand, compared with MOD, the proportion of SCFA-producing bacteria increased more in all administration groups, especially in SML-H and MET.

#### 3.4.6. Effects of SML on AI-2 content in feces of T2DM rats

**Figure 8:**
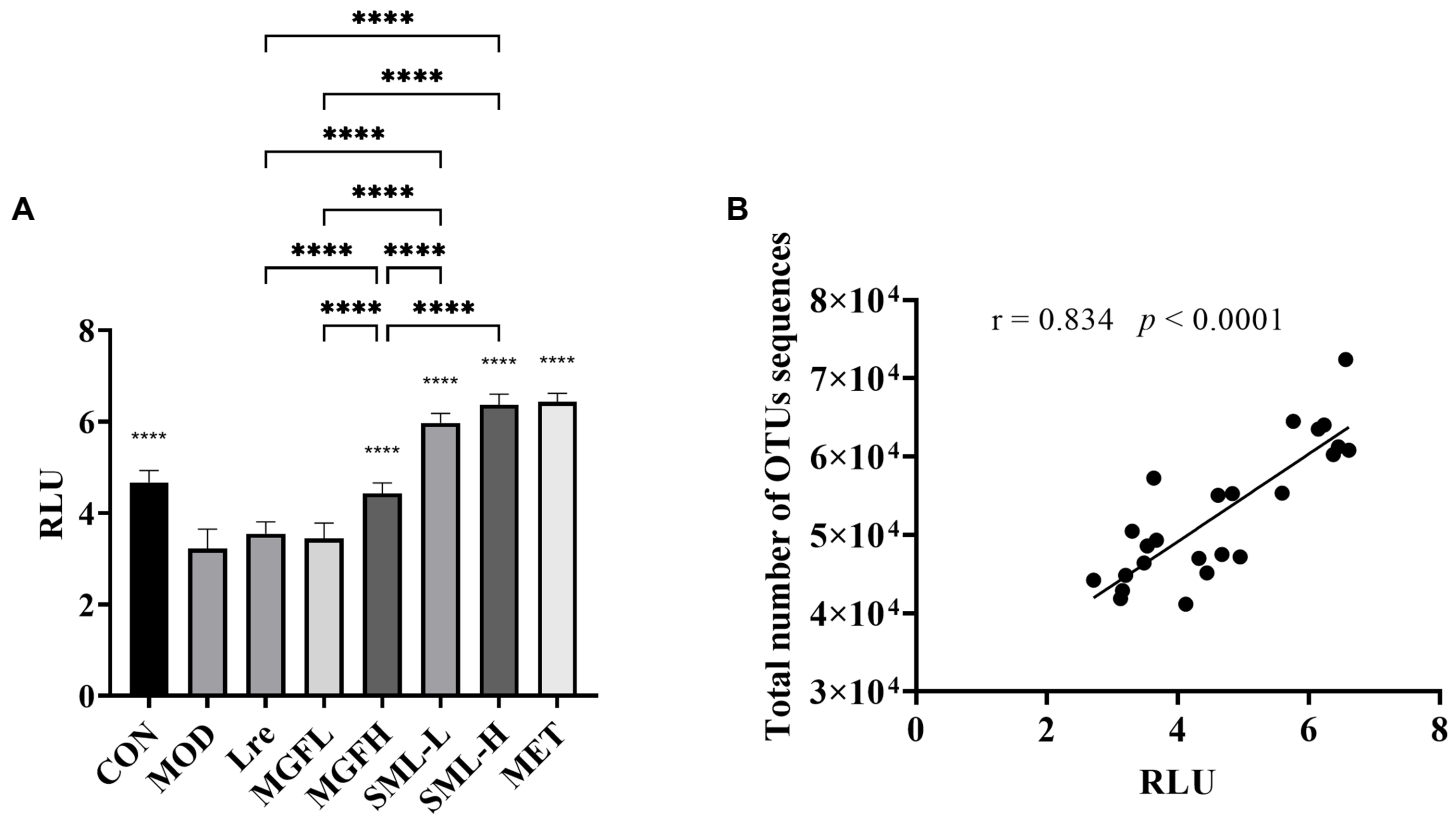
AI-2 content in feces. **(A)** was the AI-2 content in feces, ^****^p < 0.0001 compared with the MOD, and the SML and its components were compared. **(B)** was the Pearson correlation analysis between fecal AI-2 and total number of OTUs sequences.

The content of AI-2 in the feces of rats was detected. The content of AI-2 in CON, MGFH, SML-L, SML-H, and MET was significantly higher than that in MOD (*p* < 0.0001; Figure 8A). And SML had more AI-2 than its composition (Figure 8A). Importantly, the content of AI-2 in SML was higher than that of its components alone. In addition, AI-2 was positively correlated with the total number of OTUs sequences (Figure 8B).

#### 3.4.7. Effects of SML on intestinal *Lactobacillus reuteri* in T2DM rats

**Figure 9:**
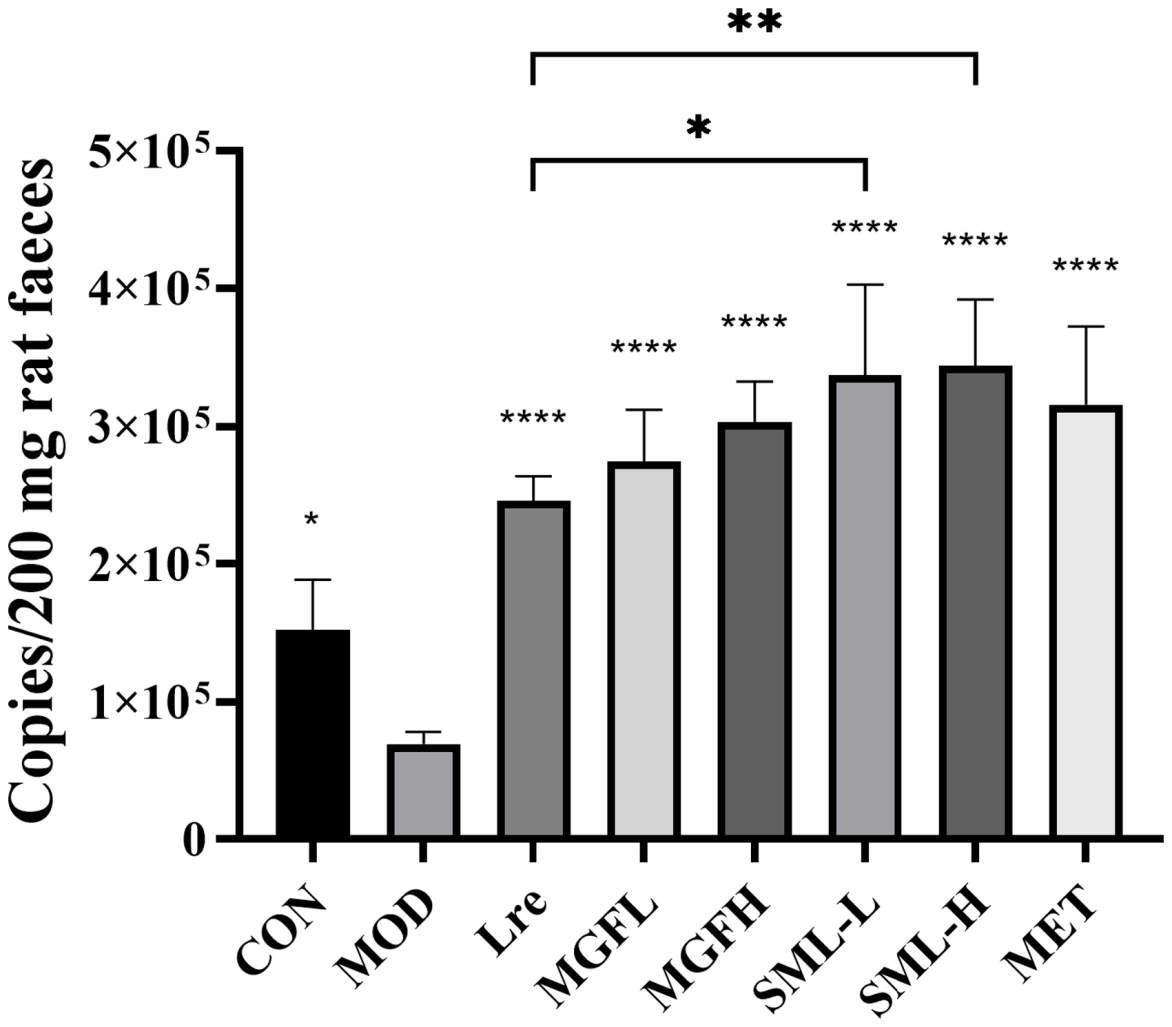
Absolute quantification results of Lactobacillus reuteri. ^*^p < 0.05, ^**^p < 0.01, ^****^p < 0.0001 compared with the MOD, and the SML and its components were compared.

The absolute quantification of *L. reuteri* in the feces was carried out. The specific detection of *L. reuteri* was performed on 11 pairs of primers, and the strong specific primer was selected for absolute quantification (Supplementary Figure S2). The absolute copy number of DNA was calculated according to the standard curve *Y* = −3.547*X* + 44.076 (*R*^2^ = 0.972). The results showed that the number of *L. reuteri* in Lre (*p* < 0.0001), MGFL (*p* < 0.0001), MGFH (*p* < 0.0001), SML-L (*p* < 0.0001), SML-H (*p* < 0.0001), and MET (*p* < 0.0001) was significantly increased (Figure 9). On the other hand, SML could significantly promote colonization of *L. reuteri* compared with Lre (Figure 9).

## 4. Discussion

Studies have shown that mangiferin can protect pancreatic β cells (Wang et al., 2014, 2018), inhibit α-glucosidase (Shi et al., 2017), improve insulin resistance (Singh et al., 2018), and promote glycolysis (Liu et al., 2018), as well as an potential mean to treat T2DM (Sun et al., 2020; Arora and Tremaroli, 2021; Zhou et al., 2022). Mangiferin had a wide range of safe concentrations, and no abnormal clinical signs or hematological changes were observed in rats after oral administration of 250–1,000 mg/kg mangiferin (Prado et al., 2015). For *L. reuteri*, a randomized, double-blind, placebo-controlled trial found that *L. reuteri* significantly reduced HbA1c and serum cholesterol (Hsieh et al., 2018). Additionally, administration of the *L. reuteri* increased insulin secretion and incretin release in humans (Simon et al., 2015). In this research, we found that SML could effectively reduce FBG and ameliorate abnormal glucose tolerance compared with its components. Furthermore, SML promoted intestinal colonization of *L. reuteri*, which may be the key to relieve T2DM.

In this study, there were significant changes in intestinal flora composition between groups. Among them, there were many undefined bacteria, which required further exploration. The abundance and diversity of CON and MOD were at the highest level, which may be due to the relatively strong effect of oral drugs on intestinal flora. According to the original data, we found fewer OTUs in the drug groups, but SML increased the proportion of probiotics, reduced the proportion of pathogenic bacteria, and had more total number of OTUs sequences, which may be the reason for the results shown in PCA and PCoA.

After SML intervention, the proportion of many probiotics increased, mainly including *Phascolarctobacterium*, *Roseburia*, *Lactobacillus acidophilus*, *Bifidobacterium pseudolongum*, *Lactobacillus murinus*, *Lactobacillus reuteri*, and *Lactobacillus johnsonii* and so on. These probiotics may play an important role in improving the pathological status of T2DM. One study found that a high level of *Phascolarctobacterium* was associated with gastrointestinal health, increased insulin sensitivity, and reduced systemic inflammation (Oluwagbemigun et al., 2021). Additionally, *Roseburia*, a kind of butyrate-producing genus, has been shown to improve glucose tolerance (Gou et al., 2021). *Lactobacillus acidophilus* could alleviate T2DM by regulating hepatic glucose, lipid metabolism, and gut microbiota in mice (Yan et al., 2019). Analogously, *Lactobacillus johnsonii* N6.2 could alleviate the development of type 1 diabetes in BB-DP rats (Valladares et al., 2010). This revealed that regulating the intestinal flora or some specific bacteria is significant for preventing and treating T2DM. In addition, the LEfSe analysis in our results showed that *L. reuteri* was labeled as a biomarker in the CON and played an important role in the intestinal tract of healthy rats. *L. reuteri* may be a good choice for alleviating T2DM.

AI-2 has a wide range of regulatory effects on bacterial flora, including proliferation, colonization, virulence, biofilm formation, and other behaviors (Miller and Bassler, 2001; Pereira et al., 2008). It has also been proposed that AI-2 in lactobacilli may represent one way of adapting to the host’s ecosystem and of interacting within the intestinal environment (Yeo et al., 2015). Another study found that mammalian epithelia can produce an AI-2 mimic activity in response to bacteria or tight-junction disruption (Ismail et al., 2016). In this study, SML promoted the proliferation and colonization of *L. reuteri* in the intestinal tract, and increased the intestinal AI-2 level. Changes in intestinal flora might be associated with AI-2, but more evidence was needed to confirm this. It is worth mentioning that intestinal AI-2 has a strong positive correlation with the total number of OTUs sequences. That said, AI-2 seems to be able to monitor gut bacteria numbers.

Short chain fatty acids play an important role in intestinal health, which enter the systemic circulation as signaling molecules and influence host metabolism. SCFAs could regulate myeloid cells and lymphocytes, promote lymphocyte production, and activate GPR43 on intestinal epithelial cells, enhance intestinal barrier function to prevent inflammatory diseases caused by bacterial invasion (Kim, 2018). Other studies have reported that SCFAs can promote the release of glucagon-like peptide-1 from enteroendocrine L cells through GPR41/43-dependent mechanisms, which highlights SCFAs as a potential target for the treatment of DM (Tolhurst et al., 2012; Everard and Cani, 2014; Kaji et al., 2014). In this study, CON had a relatively low proportion of SCFA-producing bacteria. The reason may be that the pathological state instead stimulates the host intestinal self-regulation mechanism, and SML promoted more SCFA-producing bacteria for regulation.

It has been reported that LPS induces IL-1β, IL-6, IL-8, and TNF in a TLR4-dependent manner and severely damages the survival and function of β cells (He et al., 2019). Increases in specific inflammatory markers such as IL-6 and TNF-α, which can alter insulin sensitivity by triggering insulin signaling pathways, appear to be associated with metabolic disorders (Calle and Fernandez, 2012). In addition, TNF-α (Kwon et al., 1999; Fromont et al., 2009) and IFN-γ (Zhang et al., 2011) were also associated with T2DM. We found that SML decreased LPS, ICAM-1, TNF-α, and IFN-γ, indicating that SML may relieve β cell damage, improve insulin release and sensitivity by reducing inflammatory cytokines.

## 5. Conclusion

Our previous results showed that mangiferin could significantly promote the proliferation of *L. reuteri* 1–12 *in vitro* (Meng et al., 2022). In this study, the SML showed superior therapeutic value in the treatment of T2DM rats. SML could synergistically increase the proportion of other probiotics, and play a vital role in the colonization and proliferation of *L. reuteri* in the intestinal tract. It had to be mentioned that SML can increase AI-2 in feces, where AI-2 was widely regarded as a universal signal molecule in QS, and AI-2 was strongly positively correlated with the total number of OTUs sequences. Changes in the intestinal flora may be inexplicably linked to AI-2. As a whole, SML can strongly reduce FBG, ameliorate abnormal glucose tolerance by maintaining and protecting intestinal health, and it is a good option for the treatment of T2DM.

## Data availability statement

The datasets presented in this study can be found in online repositories. The names of the repository/repositories and accession number(s) can be found at: https://www.ncbi.nlm.nih.gov/, SRP373646.

## Ethics statement

The animal study was reviewed and approved by Yunnan University of Chinese Medicine.

## Author contributions

JY and WG conceived and supervised the work. FM, FZ, MM, and YY participated in the design and implementation of the experiment. FM and FZ drafted the manuscript. QC and WW carried out data processing and analysis. HX and XL critically revised the manuscript. All authors contributed to the article and approved the submitted version.

## Funding

This research was financially supported by the Application and Basis Research Project of Yunnan China (Grants 2019IB009, 2018FF001-(005), 202001AZ070 001-037, and 202002AA100007), and Kunming International Science and Technology Cooperation Base Project (Grant GHJD-2021030).

## Conflict of interest

The authors declare that the research was conducted in the absence of any commercial or financial relationships that could be construed as a potential conflict of interest.

## Publisher’s note

All claims expressed in this article are solely those of the authors and do not necessarily represent those of their affiliated organizations, or those of the publisher, the editors and the reviewers. Any product that may be evaluated in this article, or claim that may be made by its manufacturer, is not guaranteed or endorsed by the publisher.

## Footnotes

^1^ https://cloud.majorbio.com

^2^ http://www.mothur.org/wiki/Calculators

^3^ http://huttenhower.sph.harvard.edu/LEfSe

## Abbreviations

AI-2: Autoinducer-2
DM: Diabetes mellitus
FBG: Fasting blood glucose
FINS: Fasting insulin
HF-HSD: High-fat and high-sugar diet
ICAM-1: Intercellular cell adhesion molecule-1
IFN-γ: Interferon-γ
IL-2: Interleukin-2
IL-6: Interleukin-6
LPS: Lipopolysaccharides
LuxS: S-ribosylhomocysteinase
MET: Metformin hydrochloride
OGTT: Oral glucose tolerance test
QS: Quorum sensing
SCFAs: Short chain fatty acids
SML: Synbiotic composed of mangiferin and *Lactobacillus reuteri* 1–12
STZ: Streptozotocin
T2DM: Type 2 diabetes mellitus
TNF-α: Tumor necrosis factor-α
