# Supplementary material for: Effects of the synbiotic composed of mangiferin and Lactobacillus reuteri 1–12 on type 2 diabetes mellitus rats
Source: Front Microbiol. 2023 Apr 20;14:1158652. doi: 10.3389/fmicb.2023.1158652 (PMC10157401; doi:10.3389/fmicb.2023.1158652)
Supplement: Supplementary file 1 [file Data_Sheet_1.docx]

Applied Microbiology and Biotechnology

**Effects of the synbiotic composed of mangiferin and *Lactobacillus reuteri* 1-12 on type 2 diabetes mellitus rats**

Fanying Meng^1#^ ⸱ Fan Zhang^1#^ ⸱ Meng Meng^2^ ⸱ Qiuding Chen^1^ ⸱ Yaqin Yang^1^ ⸱ Wenbo Wang^1^ ⸱ Haina Xie^1^ ⸱ Xue Li^1^ ⸱ Wen Gu^1*^ ⸱ Jie Yu^1*^

1, Yunnan Key Laboratory of Southern Medicine Utilization, College of Pharmaceutical Science, Yunnan University of Chinese Medicine, 1076 Yuhua Road, Chenggong District, Kunming, Yunnan Province, China

2, State Key Laboratory of Medicinal Chemical Biology, College of Pharmacy and Tianjin Key Laboratory of Molecular Drug Research, Nankai University, Haihe Education Park, 38 Tongyan Road, Tianjin, China

#Both authors contributed equally to this work.

*Correspondence authors at: College of Pharmaceutical Science, Yunnan University of Chinese Medicine, 1076 Yuhua Road, Chenggong District, Kunming, Yunnan Province, P. R. China.

Tel./Fax.: 13648803294 (Wen Gu), +86-871-65933303 (Jie Yu)

Email addresses: guwen1230@qq.com (Wen Gu), [cz.yujie@gmail.com](mailto:cz.yujie@gmail.com) (Jie Yu)





Figure S1. Changes in weight and food intake of rats in each group

**A** was the weight record of the rats, **B** was the food intake record of the rats.


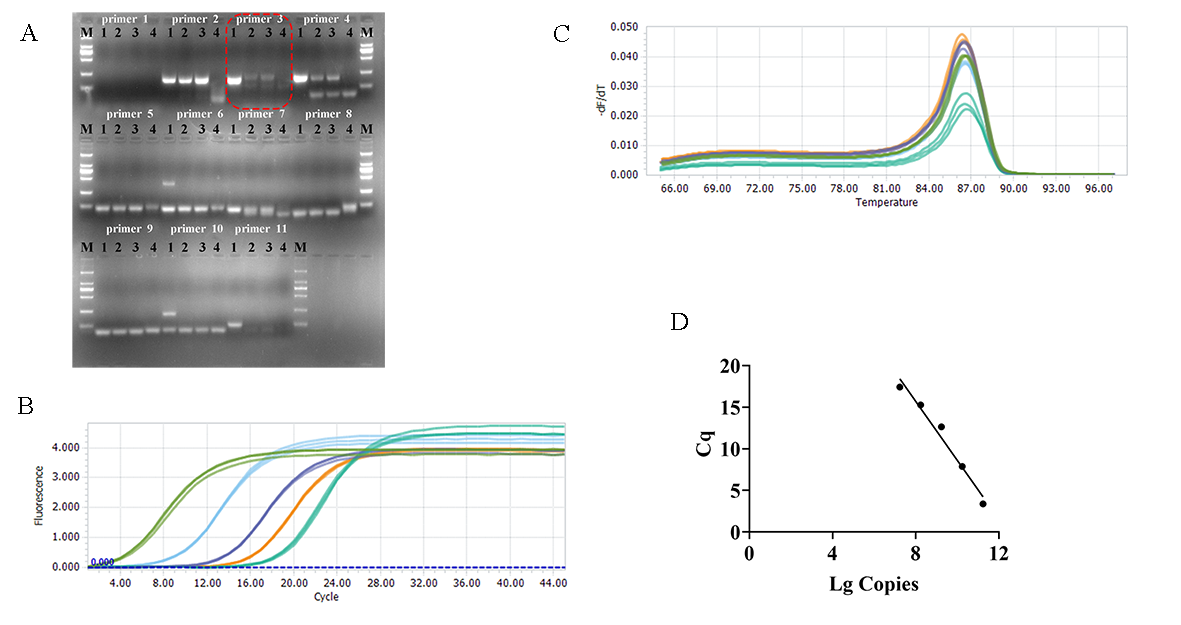


Figure S2. Absolute quantification standard curve

**A** was the screening result of specific primers, lanes 1, 2, 3, and 4 represented *L. reuteri* 1-12, *L. plantarum*, *L. faecalis 2-84,* and negative control, respectively. Each cycle 1-4 represented a pair of primers, and 11 primers were specifically screened. **B** was the amplification curve, **C** was the melting curve, and **D** was the absolute quantification standard curve.

Primer 3 had strong specificity (F 5'-GTGCTTGCACCTGATTGTC-3', R 5'-TCCCAAAGTGATAGCCAAA-3'), and the standard curve was Y = -3.547X + 44.076 (R^2^ = 0.972).

The sequence-characterized amplified region of absolute quantification results were as follows: GTGCTTGCACCTGATTGTCGATGGATCACCAGTGAGTGGCGGACGGGTGAGTAACACGTAGGTAACCTGCCCCGGAGCGGGGGATAACATTTGGAAACAGATGCTAATACCGCATAACAACAAAAGCTGCATGGTTTTTGTTTGAAAGATGGCTTTGGCTATCACTTTGGGA
